# Characterisation of newly identified vibriophages and their potential application in the biocontrol of *Vibrio parahaemolyticus* to enhance safety in aquaculture

**DOI:** 10.1016/j.virusres.2025.199664

**Published:** 2025-11-14

**Authors:** Madeline I. Petrusic, Sarah K. McLean, Enzo A. Palombo

**Affiliations:** Department of Chemistry and Biotechnology, School of Science, Computing and Emerging Technologies, Swinburne University of Technology, Hawthorn, VIC 3122, Australia

**Keywords:** Bacteriophage, *Vibrio parahaemolyticus*, Biocontrol agent, Characterisation, Phage genome, Aquaculture

## Abstract

Numerous reports have revealed that the bacterium *Vibrio parahaemolyticus* is responsible for significant economic losses in aquaculture and poses a threat to public health. In search of a sustainable biocontrol agent against *V. parahaemolyticus*, two lytic and novel bacteriophages, VpP1 and VpP2, were characterised. Both phages exhibited broad host ranges, lysing 13/16 V*. parahaemolyticus* strains. Phages were imaged by transmission electron microscopy, and analysis revealed that both phages exhibit morphology consistent with members of the class *Caudoviricetes*. Both phages inhibited the growth of their respective host bacterium with a high multiplicity of infection. All treated cultures had a substantial reduction in viability compared to non-treated cultures. One-step growth curve analysis revealed a latent period of approximately 22 min for both phages. The burst sizes were calculated as 10.2 PFU/mL, and 7.3 PFU/mL for VpP1 and VpP2, respectively. Stability determination assays revealed both phages could withstand a broad salinity range (1 to 20 %), pH levels (3.0–10.0), temperatures (-20 to 60 °C) and sodium hydrochlorite levels (3 to 10 mg/mL). Phages VpP1 and VpP2 could withstand direct UV light for 4 and 5 min, respectively. Next-generations sequencing revealed that VpP1 (63,510 bp) and VpP2 (64,298 bp) did not contain known virulence, antibiotic-resistance or lysogenic genes, highlighting their capability to be used as biocontrol agents. Phylogenetic analysis based on the major head protein revealed that VpP1 and VpP2 were categorised into the newly described *Mardecavirus* genus. The results suggest VpP1 and VpP2 have several beneficial qualities for future use as biocontrol agents. Further study is still required to determine their efficiency within food applications.

## Highlights

• Novel vibriophages VpP1 and VpP2 had broad host ranges infecting 13/16 *Vibrio* strains tested.
• Vibriophages could withstand various environmental stresses (pH, sodium hypochlorite, salinity, thermal, and UV).
• Toxin, tRNA, lysogenic and virulence genes were absent in both vibriophages.

## 1 Introduction

Foodborne disease is a significant public health concern worldwide. Ready-to-eat foods are increasingly implicated in disease outbreaks, and *Vibrio* is a pathogen of growing concern to the seafood industry (Ina‐Salwany et al., 2018). Pathogenic *Vibrio* species that cause foodborne diseases are known to rapidly reproduce and produce toxins, thus increasing the need to find solutions to control these bacteria in susceptible foods (Jones, 2014).

*Vibrio parahaemolyticus* is a Gram-negative, halophilic bacterium, typically found in warm coastal waters. Infection by the zoonotic pathogen generally presents mild symptoms such as vomiting, diarrhoea and stomach cramps in humans. However, the more vulnerable population are at increased risk of severe, potentially life-threatening conditions (Manchanayake et al., 2023). While in aquatic animals, particularly in shrimp, the bacterium can cause Acute Hepatopancreatic Necrosis Disease (AHPND) (Cao et al., 2021).

Shellfish are particularly vulnerable to bacterial contamination, as they concentrate microorganisms from surrounding waters while filter feeding. Illnesses from these species can arise from consuming raw or undercooked seafood. Human infections caused by *V. parahaemolyticus* are surging, with numerous studies linking this increase to global warming (Baker-Austin et al., 2013; Kokashvili et al., 2015; You et al., 2021). Antibiotic resistance in *V. parahaemolyticus* has become a significant concern due to extensive antibiotic use. High resistance levels, especially to ampicillin, are frequently observed across strains from various environments (Elmahdi et al., 2016; Jiang et al., 2019; Mohamad et al., 2019). The widespread occurrence of multidrug resistance complicates treatment and infection control efforts. Furthermore, resources for controlling *Vibrio* in the aquaculture industry remain limited. Disinfectants are considered unsafe, antibiotics contribute to the rise of drug-resistant bacteria, and standard post-harvest processes can impact the sensory characteristics of seafood. One alternative gaining traction is the use of bacteriophages, which offers a 'clean and green' solution compared to antibiotics and traditional sanitation methods (Kim et al., 2021; Yen et al., 2017; Zhang et al., 2018).

Bacteriophages (phages), the most abundant viruses on earth, naturally prey on bacteria by hijacking their cellular machinery and causing cell lysis. Initially explored for treating bacterial infections, phages were overshadowed by antibiotics. However, rising antibiotic resistance has renewed interest in phage therapy, which is eco-friendly, highly specific to bacterial hosts, and harmless to marine ecosystems (Greer, 2005; Picozzi et al., 2021). Phage biocontrol has proven to show promising results for reducing and eliminating bacteria on various food products (Figueiredo and Almeida, 2017; Ishaq et al., 2020; Kuek, 2024; McLean et al., 2013). In aquaculture, numerous studies have reported successful application of phages in controlling bacteria populations. For instance, a six-phage cocktail reduced the growth of *V. parahaemolyticus* in manila clams by 2.1 log CFU/mL (Chang et al., 2016). Oyster larvae infected with a lethal dose of *V. coralliilyticus*, achieved a 91 % survival following treatment with a phage-cocktail (Richards et al., 2021). In a similar study, Droubogiannis and Katharios (2022) significantly improved the survival of larvae infected with *V. harveyi* for up to five days post-phage exposure (Droubogiannis and Katharios, 2022).

In this study, novel phages VpP1 and VpP2 with lytic ability specific to *V. parahaemolyticus* strains underwent biological and genomic characterisation. The result of this research suggests a process for controlling future outbreaks of *V. parahaemolyticus* during seafood production.

## 2 Materials and methods

### 2.1 Bacterial strains and growth conditions

Phage lytic ability against sixteen *Vibrio* strains was tested (Table 1)*.* All overnight bacterial cultures were grown aerobically in a shaking incubator at 30 °C and 190 rpm in Tryptic Soy Broth (TSB) (Thermo Fisher Scientific Inc.).

**Table 1:** Vibrio spp. strains and characteristics.

Bacterial strain	Abbreviation	Source
*Vibrio parahaemolyticus*		
AUSMDU00060446	Vp60446	MDU PHL
AUSMDU00066674	Vp66674	MDU PHL
AUSMDU00060375		MDU PHL
AUSMDUO0060416		MDU PHL
AUSMDU00060385		MDU PHL
AUSMDU00059795		MDU PHL
AUSMDU00066821		MDU PHL
AUSMDU00060396		MDU PHL
AUSMDU00059440		MDU PHL
AUSMDU00060086		MDU PHL
L102 V5		Monash University
AP216		AusPhage
AP218		AusPhage
AP230		AusPhage
*Vibrio vulnificus*		
27,652 #8		Swinburne University
27,652 #9		Swinburne University

MDU PHL: Microbiological Diagnostics Unit Public Health Laboratory, University of Melbourne.

AusPhage: AusPhage Pty Ltd, Townsville, Queensland.

### 2.2 V*. parahaemolyticus* antibiotic sensitivity

*V. parahaemolyticus* AUSMDU00060446 (Vp60446) and AUSMDU00066674 (Vp66674) were tested against five commercial antibiotics using the Kirby-Bauer disk diffusion assay method (Yano et al., 2014). Bacterial lawns were prepared on TSB agar using a sterile cotton swab and antibiotic disks (Ampicillin, Chloramphenicol, Ciprofloxacin, Gentamicin, Imipenem and Tetracycline) (ThermoFisher, Australia) were applied. Plates were incubated for 24 h at 30 °C and inhibitions zones were measured and categorised into sensitive, intermediate or resistant according to the Clinical and Laboratory Standards 2012 standards (CLSI, 2012).

### 2.3 Vibriophage host range assay

VpP1 and VpP2 were previously isolated by AusPhage Pty Ltd (Townsville, Queensland) (unpublished data). Sixteen *Vibrio* strains were tested as potential host bacteria for phages the VpP1 and VpP2 described in this report. Host specificity was completed by applying the double agar overlay method with an overnight host *Vibrio* strain, and either phage was spotted on the agar. Plates were incubated overnight at 30 °C, and host bacteria were chosen by observing the clearest plaques formed (Chen et al., 2017).

### 2.4 Efficiency of plating

Bacterial strains sensitive to either VpP1 or VpP2 phages during the spot tests described in Section 2.3 were selected for efficiency of plating (EOP), as described by Kutter (2009). Plaque assays were conducted against the subjected strains using the soft molten agar overlay method. The EOP values were ranked as ‘High EOP’ (EOP ≥ 0.5), ‘Medium EOP’ (0.1 ≤ EOP ≤ 0.5), ‘Low EOP’ (0.001 < EOP < 0.1), and ‘No EOP’ (EOP ≤ 0.001) (Kutter, 2009).

### 2.5 Vibriophage morphology

A Joel-JEM 2100 Transmission Electron Microscope (TEM) was used to observe the morphology of VpP1 and VpP2. Five µL of phage stock (10^8^–10^10^ PFU/mL) were added onto copper grids with carbon film. The grids were negatively stained with 5 µL of 0.2 % uranyl acetate, followed by blotting of the sample with filter paper to remove excess stain. Grids were left to air dry at room temperature before being viewed under the electron microscope. Images were analysed with ImageJ software (National Institutes of Health, USA). At least five distinct phage particles were measured to determine the size average.

### 2.6 Determination of multiplicity of infection

The multiplicity of infection (MOI) for VpP1 and VpP2 was performed based on the method previously described by Benala et al. (2021). Tested MOIs ranged between 0.001 to 1000,000. Briefly, 150 µL of TSB broth was added as the blank, the positive control was 50 µL of either phage, and 150 µL of the exponential phase bacteria (10^4^ CFU/ml) was the negative control. Phage stocks were serially diluted in SM buffer from 10^9^ to 10^1^ PFU/mL, and 50 µL was added to each well to achieve an MOI from 0.001 to 1 million. The absorbance (OD_600_) was read using the FLUOstar® Omega Plate Reader (BMG LabTech) at 30 °C for 24 h, measuring intervals every 30 min and programmed to shake (linear) 10 s before each cycle was measured (Benala et al., 2021).

### 2.7 One-step growth curve for vibriophages

A one-step growth curve was performed to determine the phage’s latent period and burst size. *Vibrio* host strains were adjusted to reach 10^8^ CFU/mL. One hundred microliters of phage (10^7^ PFU/mL) was combined with 900 µL of the respective host strain and added to the shaking incubator for 5 min, set to 30 °C and 190 rpm. To synchronise the viral infection, the phage-bacteria suspension was centrifuged for 5 min, adjusted to 4 °C and 10,000 x *g*, and resuspended in 1 mL of TSB. The suspension was added to 9.9 mL of TSB and incubation was continued in the shaking incubator under the same conditions for one hour, with sampling taken at various time intervals. The soft agar overlay method was applied after each time point was sampled and plates were incubated overnight at 30 °C. Experiments were performed in triplicate and the average of each time interval was used for the final growth curve (Kim et al., 2021)

### 2.8 Bacteriophage stability under different environmental conditions (thermal, pH, salt, UV and sodium hypochlorite)

To assess phage stability at various temperatures, phages were diluted in SM buffer to 10⁶ PFU/mL and incubated for one hour at −20 °C, 10 °C, 20 °C, 40 °C, 60 °C and 80 °C. A sample maintained at 4 °C served as the negative control, reflecting standard storage conditions (Kim et al., 2021).

For pH stability, phages (10^6^ PFU/mL) were added to SM buffer and adjusted to different pH conditions of 2, 3, 4, 6, 7.5, 9, 10 and 12. A pH of 7.5 served as the control, reflecting the standard pH of SM buffer. Adjustment of pH levels was made using 1 M HCL and 1 M NaOH. Phages were incubated for one hour at 30 °C (Kim et al., 2021).

For salt stability, phages (10^6^ PFU/mL) were added to SM buffer, adjusted to different sodium chloride (NaCl) concentrations of 1 %, 2 %, 5 %, 10 %, 15 % and 20 %. Phage solution with no added NaCl acted as the control. Phages were incubated for one hour at 30 °C (Cubo et al., 2020).

Phages were exposed to UV light using the method described by Ramirez et al. (2018), with minor modifications. Briefly, 100 µL of phages (10^6^ PFU/mL) were added to sterile Petri dishes and placed 10 cm under a UV lamp (Camag, UV-betrachter) (λ= 254 nm) for 0.5, 1, 2, 3, 4 and 5 min. A non-exposed phage sample operated as the control (Ramirez et al., 2018).

To test phage stability in 4 % sodium hypochlorite (Chemwell Products, Victoria), the solution was diluted in dH_2_O to a concentration of 0.5 %. This was further adjusted to reach levels 10, 8, 5 and 3 ppm. A control sample of phages without the solution was included. Briefly, 250 µL of the various sodium hypochlorite levels and 250 µL of phage were combined and left for one hour at 20 °C before using the soft agar method to determine the concentration of infective phages (Lingga et al., 2020). All phage titres for the above section were determined using the soft agar overlay method.

### 2.9 Measuring the frequency of bacteriophage-insensitive mutants (BIM)

The frequency of emerging BIMs was determined following the method described by Bai *et al*. (2019). Briefly, 500 µL of overnight bacterial cultures (10⁶ CFU/mL) were mixed with 500 µL of the corresponding phage (10⁸ PFU/mL) and incubated at 30 °C on a shaking platform at 190 rpm for 10 min. The mixture was then serially diluted in phosphate-buffered saline (PBS), and 100 µL was plated on TSB agar. Plates were incubated overnight. BIMs obtained from single-phage treatments were sub-cultured and tested for susceptibility to other vibriophages using spot assays (Bai et al., 2019).

### 2.10 Analysis of phages using next-generation sequencing

Bacteriophage DNA was extracted using the Phage DNA Isolation Kit (Norgen Biotek Corp., Canada, Product #46,800, 46,850). Nanodrop One UV–Vis was used to determine the concentration of phage nucleic acid extraction. Nextera XT DNA construction kit was used and the library prep were constructed according to the manufacturer’s instructions, with the following parameters; DNA purification with Axygen® AxyPrep MAG PCR clean-up kit (Corning, USA) using 2 V before library construction, input DNA mass (0.5 ng), libraries amplified with 16 cycles of PCR, libraries purified and subjected to double-sized selected using 0.6 V and 0.8 V of Axygen® AxyPrep MAG PCR clean-up kit (Corning, USA) (Illumina Inc., 2023).

Phage genomes were assembled using Unicycler v0.4.7, a platform that uses short reads to construct fragmented but accurate contigs (Wick et al., 2017). The *de-novo* assembly method was applied to construct phage genomes from large DNA fragments with no reference strain. Phage genomes were annotated using Pharokka v1.3.2 (Bouras et al., 2023). To generate phages genome map, the contig files were uploaded to Proksee and each ORFs (< 800 bp) that was found on the forward strand (ORF(+)) was located on SnapGene® Viewer software (www.snapgene.com) and added to NCBI’s ORFfinder (https://www.ncbi.nlm.nih.gov/orffinder/) tool to determine whether there is a known protein function.

Phage major head proteins were added to BLASTn to search for similar phage sequences in GenBank. To analyse the evolutionary relationships of phages, closely related phage sequences were added to MEGA software version 11 for phylogenetic tree construction (Tamura et al., 2021).

### 2.11 Statistical analysis

All experiments for this study were completed in technical triplicate and all data were presented as mean ± standard deviation. Statistical significance was obtained with a *p*-value less than or equal to 0.05.

## 3 Results

### 3.1 Bacteriophage host range assay and efficacy of plating

Novel bacteriophages specific for *V. parahaemolyticus*, VpP1 and VpP2, were isolated in Vietnam (AusPhage Pty Ltd) and tested on their lytic ability against 16 *Vibrio* strains (13 V*. parahaemolyticus* strains and three *V. vulnificus* strains) (data not shown). The *Vibrio* strains used in the host range assay were sourced from the Microbiological Diagnostics Unit Public Health Laboratory (MDU PHL), and most were environmental isolates obtained from infected oysters. This selection was intentional to reflect real-world relevance, potentially for applications in shellfish aquaculture. VpP1 produced the clearest plaque on a *V. parahaemolyticus* Vp66674 lawn and was chosen as the host bacteria in further experiments, while Vp60446 was selected as the host for VpP2. Both phages infected just over 80 % of the tested strains (13/16). However, they only produced plaques for *V. parahaemolyticus* strains; the lack of lysis in *V. vulnificus* strains supports species specificity.

Bacterial strains that produced clear plaques were tested for their efficiency of plating (EOP) (Table 2). The EOP was measured by comparing titres between *Vibrio* test strains and chosen host bacteria as a reference strain. VpP1 had high lytic efficiency for two of the seven (28 %) *V. parahaemolyticus* isolates, three (42 %) isolates had medium lytic efficiency, one (14 %) isolate had low lytic efficiency, and one (14 %) had inefficient lytic ability. VpP2 demonstrated high, medium and low lytic efficiency against 3 (42 %), one (14 %) and 2 (28 %) strains, respectively. *V. parahaemolyticus* isolates that produced inefficient EOP values indicate that phages were able to inhibit bacterial growth during spot testing but could not lyse the bacteria.

**Table 2:** EOP values with ranking order for VpP1 and VpP2 and antibiotic resistance profile of host strains (CLSI guidelines).

V. parahaemolyticus	VpP1			VpP2		Antibiotic resistance
	EOP value	EOP ranking		EOP value	EOP ranking	
Vp60446^a^	0.17		++	1	**+++**	AMP
Vp66674^a^	1		+++	0.004	+	AMP, CN, CIP
AUSMDU00060375	0.03		+	0.11	++	
AUSMDUO0060416	0.1		++	0.06	+	
AUSMDU00060385	0.74		+++	0.43	+++	
AUSMDU00059795	0.17		++	0.69	+++	
AUSMDU00060396	< 0.0001		-	< 0.0001	-	

High: (+++) EOP ≥ 0.5, Medium: (++) 0.1 ≤ EOP ≤ 0.5, Low: (+) 0.001≤ EOP ≤ 0.1, Inefficient: (-) EOP ≤ 0.001.

a Host bacterium.

### 3.2 V*. parahaemolyticus* antibiotic sensitivity

The two *V. parahaemolyticus* host strains, Vp66674 and Vp60466, were tested for their antibiotic-resistant phenotype to confirm the vibriophages used for this study can effectively reduce multidrug-resistant strains. Out of the six antibiotics tested, Vp60446 was resistant to ampicillin, while Vp66674 was resistant to ampicillin, gentamicin and ciprofloxacin (Table 2.)

### 3.3 Bacteriophage propagation and morphological characteristics

**Fig. 1:**
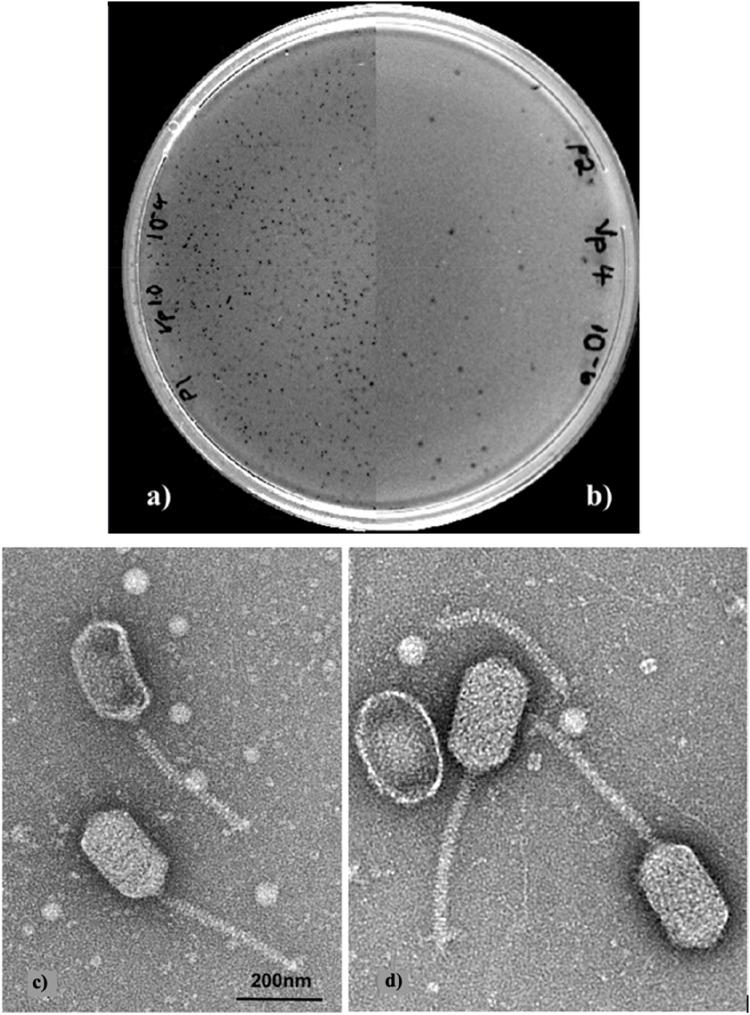
Plaques produced by vibriophage **a)** VpP1 and **b)** VpP2. Transmission electron microscopy images of vibriophage **c)** VpP1 and **d)** VpP2. Scale bar 200 nm.

Both phages produced small plaques, varying between 0.5–0.7 mm in diameter. The host bacteria determined for VpP1 was *V. parahaemolyticus* isolate Vp60446 (Fig. 1a), which produced the greatest lytic ability. The host bacterium for VpP2 was Vp66674 (Fig. 1b).

Transmission electron microscopy (TEM) images revealed that both phages possessed elongated icosahedral capsid heads and non-contractile tails (Fig. 1c and 1d). Characteristics of these vibriophages are shown in Table 3. These structural features are consistent with those historically associated with the former *Siphoviridae* family, which was part of the now-obsolete order *Caudovirales* (Turner et al., 2023)*.* While such morphology provides useful structural context, it no longer serves as a basis for formal classification under the current International Committee on Taxonomy of Viruses (ICTV) guidelines.

**Table 3:** Characteristics of the vibriophages in this study. Data presented as an average of five phages measured.

Phage	Head (nm)		Tail (nm)	
	Width	Length	Width	Length
VpP1	57.5 ± 1.2	102.7 ± 2.4	12.4 ± 1.6	155 ± 6.3
VpP2	56.7 ± 2	100.9 ± 2.7	12.6 ± 1.3	155 ± 7.3

### 3.4 Inhibition of bacterial growth after vibriophages treatment at different multiplicity of infections (MOI)

**Fig. 2:**
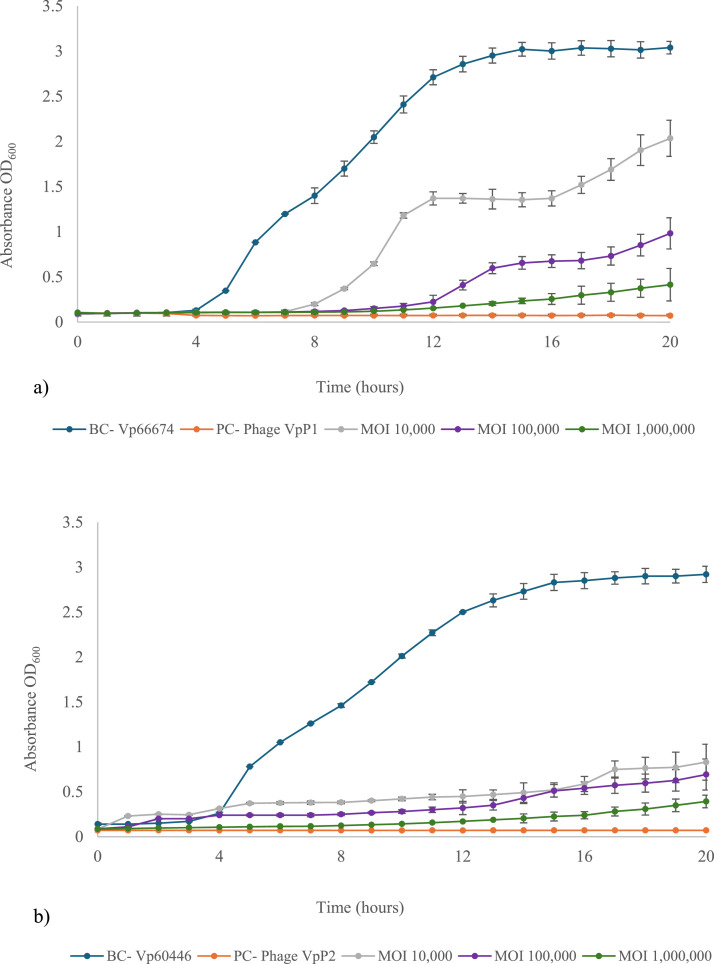
Growth of host bacteria after vibriophage treatment at varying MOI for vibriophage **a)** VpP1 and **b)** VpP2. BC- bacteria control; PC- phage control.

The ability of phages to control *V. parahaemolyticus* strains was tested over 20 h to determine their optimal MOI. Narrow range MOI levels (0.01 to 1000) were also tested; however, both phages were unable to control bacterial growth for longer than 8 h (data not present). Data from host bacteria that had no growth inhibition (MOI 0) presented a standard sigmoid curve, while broad-range MOI levels of 10,000, 100,000 and 1000,000 exhibited a stronger ability to control the host bacterium during the experimental period. Growth of Vp66674 was prevented by VpP1 at an MOI of 10,000 and 100,000 until 8 and 12 h, respectively, before Vp66674 began to grow (Fig. 2a). VpP2, at MOIs of 10,000 and 100,000, was able to suppress the growth of Vp60446, maintaining absorbance values below 0.50 (Fig. 2b). The optimal MOI is defined as the lowest value that minimises bacterial growth (Abedon, 2016). For both vibriophages, this corresponded to the highest MOI tested, namely 1000,000. At this MOI, VpP1 effectively inhibited the growth of Vp66674 for approximately 13 h before host bacteria began to regrow. Similarly, VpP2 maintained suppression of its host bacterium for the same duration, followed by a modest increase in bacterial growth.

### 3.5 One-step growth curve analysis

**Fig. 3:**
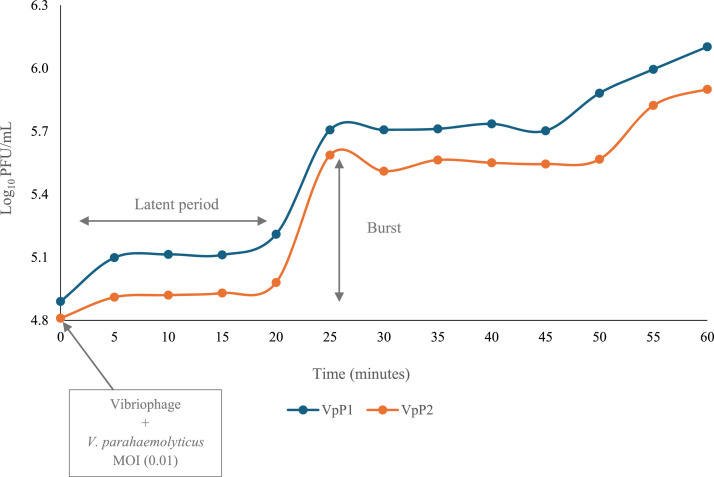
One-step growth curve for VpP1 on V. parahaemolyticus strain Vp60446 and VpP2 on strain V. parahaemolyticus Vp66674. Data points represent the mean values from three independent replicates; error bars indicate the standard deviation of the means.

One-step growth curve experiments are used to determine the life cycle of a phage. The growth curve in Fig. 3 demonstrates very similar burst sizes and latent periods for both VpP1 and VpP2. The eclipse period lasted approximately 20 min for both vibriophages. VpP1 had a latent period of 22.5 min, while VpP2 was 22 min. The first burst period lasted 10 min and released 10.2 PFU/cell for VpP1 and 7.3 PFU/cell for VpP2. Following the first burst at 30 min, both vibriophages were stable until their second burst at 50 min (Table 4).

**Table 4:** Latent period and burst size of VpP1 and VpP2 based on the one-step growth curve.

Phage	Latent period (min)	Burst size (PFU/cell)
VpP1	22.5	10.2
VpP2	22	7.3

### 3.6 Vibriophage stability at a range of different salt, pH, thermal, UV and sodium hypochlorite concentrations

**Fig. 4:**
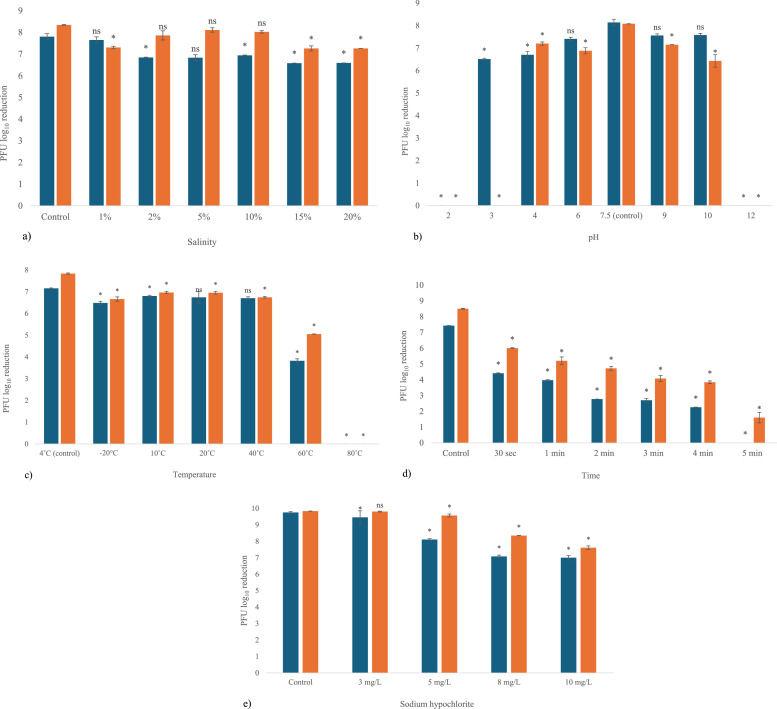
Stability of  VpP1 and  VpP2 at various **a)** salinities (1 %, 2 %, 5 %, 10 %, 15 %, 20 %), **b)** pH levels (2,3, 4, 6, 7.5, 9, 10, 12), **c)** temperatures (−20 °C, 4 °C, 10 °C, 20 °C, 40 °C, 60 °C, 80 °C) **d)** times under UV (0.3, 1, 2, 3, 4, 5) and **e)** sodium hypochlorite concentrations (3 mg/L, 5 mg/L, 8 mg/mL, 10 mg/mL). All experiments were conducted for one hour, with the exception of the UV stability assay. Data points represent the mean values from triplicate samples; error bars indicate the standard deviation of the means. * indicates a statistical significance *p* < 0.05, ns signifies no statistical significance *p* > 0.05.

Fig. 4a illustrates the stability of VpP1 and VpP2 across varying salt concentrations. VpP1 (10^8^ PFU/mL) showed a gradual decline with increasing salinity, reaching a maximum reduction of 1.2 log_10_ PFU/mL at 20 % NaCl (*p* < 0.05). VpP2 was comparatively more resilient, with its greatest reduction being 1.0 log_10_ PFU/mL at 20 % (*p* < 0.05). Statistically significant reductions were observed at 2 % and 10 % for VpP1, at 1 % for VpP2, and at 15 % and 20 % for both vibriophages.

Fig. 4b shows that both vibriophage became suspectable after one hour of exposure to extreme pH levels (pH 2 and 12). VpP1 remained infective at a pH of 3, with a decrease of 2 log_10_ PFU/mL (*p* < 0.05). No significant decrease was observed at pH 6, 9 and 10 (*p* > 0.05). Whereas, VpP2 exhibited significant reductions at all tested pH levels (*p* < 0.05).

Fig. 4c indicates that VpP1 titres remained stable at 20 °C and 40 °C (*p* > 0.05), but declined significantly at higher temperatures (*p* < 0.05), with a maximum reduction of 60 %, corresponding to 3.8 log_10_ PFU/ml. VpP2 was less sensitive, showing a 43.1 % decrease to 5 log_10_ PFU/ml at 60 °C (*p* < 0.05). Both vibriophages were inactive at 80 °C (*p* < 0.05).

Fig. 4d shows that UV exposure caused rapid inactivation of both vibriophages. Within 30 s, titres of VpP1 and VpP2 decreased by 3 and 2.5 log_10_ PFU/mL, respectively (*p* < 0.05). VpP1 became inactive after 5 min. VpP2 showed a gradual decline of 0.5–0.7 log_10_ PFU/mL between 1 and 4 min, followed by a further reduction of 2.2 log_10_ PFU/mL, but remained infective at 5 min (*p* < 0.05).

Fig. 4e illustrates that both vibriophages remained infective across all tested sodium hypochlorite concentrations. At 5 mg/mL, VpP1 and VpP2 titres decreased by 1.6 and 0.3 log_10_ PFU/mL, respectively (*p* < 0.05). Both phages continued to be infective at 10 mg/mL sodium hypochlorite (*p* < 0.05).

### 3.7 Evaluation of bacteriophage insensitive mutants (BIM) emergence and their susceptibility to other vibriophages

**Fig. 5:**
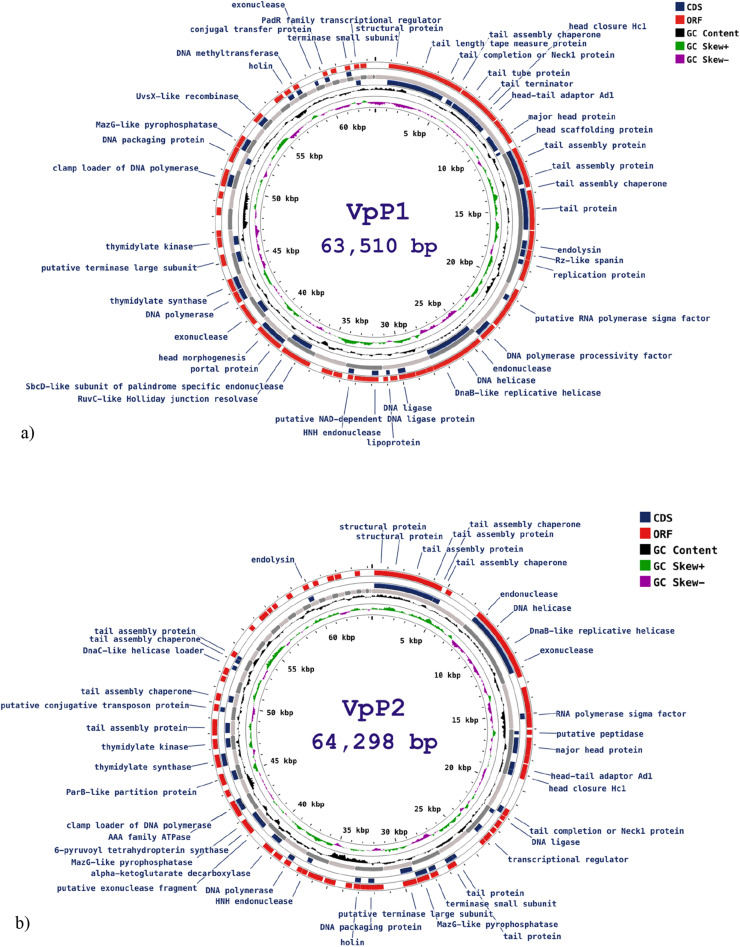
Genomic structure of vibriophage **a)** VpP1 and **b)** VpP2. Hypothetical proteins with unknown functions are not displayed. The figure was visualised and annotated using Proksee software. Map circle (beginning from the outside): ORFs in forward and reverse direction (red), CDS (navy), contig (light and dark grey), GC content (black) and GC Skew+ and Skew- (green and purple).

BIM frequency was assessed by sub-culturing colonies that appeared following single-phage treatment. These colonies displayed altered morphologies compared to the parental strain. To quantify BIM emergence, both vibriophages were tested at a multiplicity of infection (MOI) of 100 against their respective bacterial hosts. Cross-infectivity was evaluated by exposing BIMs derived from VpP1 to VpP2, and *vice versa*, to test whether resistance was specific to the original phage or extended to others (Fig. 5).

### 3.8 General features of vibriophages genome analysis using next-generation sequencing

**Fig. 6:**
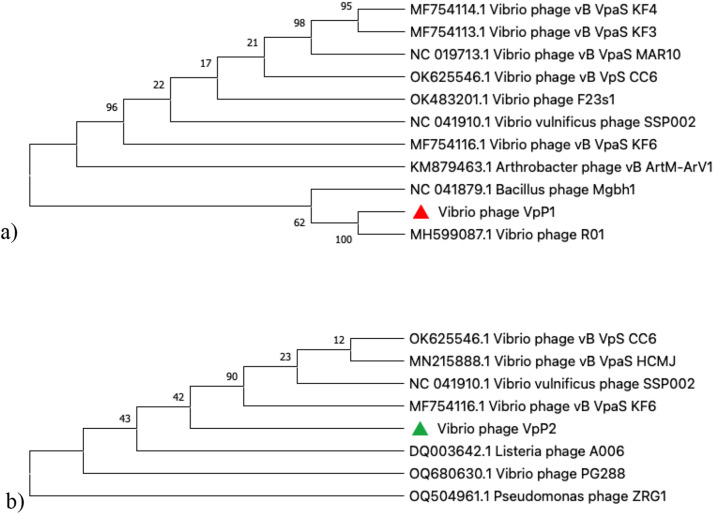
Phylogenetic tree based on major head protein of vibriophage **a) ▲** VpP1 **b) ▲** VpP2 and 17 members of the Caudoviricetes order. Phylogenetic tree was generated using the neighbour-joining method with 1000 bootstrap replicates and analysis was completed using the MegaX software.

VpP1 and VpP2 were assembled using Unicycler v0.4.7, and their double-stranded genomes were assembled in Proksee. *De-novo* assembly generated 78 contigs for VpP1, a linear genome of 63,510 bp and a *G* + *C* content of 49 %. There were 82 coding sequences, 45 of which were known vibriophage functional proteins, 37 were hypothetical proteins, and 94 were ORFs. No ORF(+) (>800 bp) searched had known protein functions. The known protein functions can be categorised into those related to phage DNA replication, cell lysis, structural proteins and packaging (Fig. 6a). The presence of endolysin, Rz-like spanin and UvsX-like recombinase are genes associated with virulent phages. No lysogenic genes, toxin genes or antibiotic resistance genes were found in the VpP1 genome.

*De-novo* assembly generated 111 contigs for VpP2, a linear genome of 64,298 bp and a *G* + *C* content of 49 %. A total of 74 CDCs were found, 42 with known protein functions and 32 were hypothetical proteins. VpP2 had 85 ORFs; however, none over 800 bp had known protein functions when searched in the NCBIs ORF-finder. The known protein functions of VpP2 were also categorised into DNA replication, cell lysis, structural proteins and packaging, seen in Fig. 6b The VpP2 genome does not present any genes associated with lysogenic phage, as well as no tRNA-encoding, antibiotic-resistant and toxin genes.

### 3.9 Phylogenetic tree and taxonomy classification

The phylogenetic tree of VpP1 and VpP2 were constructed based on the major head proteins. This was done so to determine the evolutionary relationships among various vibriophages, by comparing similar sequences with BLASTn. Ten phages with similar sequences were used to construct the phylogenetic tree for VpP1 (Fig. 7a). According to the phylogenetic analysis, VpP1 was closely related to *Vibrio* phage R01 (MH599087) with a 90.54 % pairwise sequence identity. VpP2 did not have as many close relationships as VpP1. Seven phages with similar sequences were used to construct Fig. 7b *Vibrio* phage vB_VpaS_KF6 (MF754116.1) had an 83.68 % pairwise sequence identity with VpP2. The major head protein belonging to *Vibrio* phage vB_VpaS_KF6 had 99.82 % and 99.64 % pairwise identity similarity with VpP1 and VpP2, respectively.

## 4 Discussion

Phage-based solutions present a promising and sustainable alternative to antibiotics in aquaculture, addressing the growing concern of antimicrobial resistance. Current research highlights their efficiency in enhancing marine organism health and their potential role in improving food safety by mitigating bacterial contamination during seafood processing.

In this study, two *Vibrio*-specific bacteriophages, VpP1 and VpP2, were evaluated for their potential as biocontrol agents in aquaculture. A broad host range, defined as a phage’s ability to infect multiple strains within the same species, is a desirable trait for effective pathogen control (Fong et al., 2021). Host-range analysis revealed that VpP1 and VpP2 exhibited lytic activity against 13 out of 16 (81 %) tested *V. parahaemolyticus* strains. The two host strains, *V. parahaemolyticus* Vp66674 and Vp60446, demonstrated resistance to three antibiotics commonly used in aquaculture, highlighting their contribution to the growing issue of antibiotic-resistant bacteria (Bondad-Reantaso et al., 2023). The ability of VpP1 and VpP2 to target and suppress these multi-drug resistant strains highlights their potential value as sustainable alternatives to antibiotics in aquaculture.

Transmission electron microscopy (TEM) revealed that VpP1 and VpP2 possess elongated icosahedral capsid heads and non-contractile tails, structural features that were historically associated with the now-obsolete family Siphoviridae. The ICTV has recently revised its classification system for tailed double-stranded DNA (dsDNA) phages, eliminating morphology-based families such as Siphoviridae, Myoviridae, and Podoviridae, along with the order Caudovirales. Under this updated framework, morphology no longer determines taxonomic placement. Instead, classification is based solely on genomic data (Turner et al., 2023). In this study, genomic analysis has placed VpP1 and VpP2 within the class *Caudoviricetes*, which includes tailed dsDNA phages with icosahedral capsids. These findings are consistent with TEM imaging and genomic data reported in other vibriophage studies (Benala et al., 2023; Chaichana et al., 2025; Zhang et al., 2024). Further phylogenetic and comparative genomic analysis revealed that VpP1 shares close similarity with eight *Vibrio*-specific phages, as well as targeting *Bacillus* and *Arthrobacter*. According to the phylogenetic analysis, VpP1 was closely related to *Vibrio* phage R01 (MH599087) with a 90.54 % pairwise sequence identity. The major head protein of *Vibrio* phage vB_VpaS_KF6 (MF754116.1) exhibited 99.82 % and 99.64 % pairwise identity with VpP1 and VpP2, respectively. However, this high similarity relates specifically to a highly conserved structural protein, which is commonly used in phylogenetic analyses due to its evolutionary stability (Tétart et al., 2001). Importantly, the overall genome-wide pairwise identity between VpP2 and vB_VpaS_KF6 is 83.68 %, indicating substantial genomic divergence beyond conserved regions. As outlined by Turner et al. (2023), a nucleotide identity threshold of ≤95 % is required to define a new phage genus. In accordance with ICTV guidelines and Turner et al. (2023), the overall nucleotide identity below 95 % supports the classification of VpP1 and VpP2 within a distinct genus, reinforcing their placement within the class *Caudoviricetes* and assignment to the genus *Mardecavirus* (Turner et al., 2023).

Understanding the multiplicity of infection (MOI) is essential for the practical application of phages in aquaculture (Benala et al., 2021). In this study, MOI values ranging from 0.001 to 1000,000 were tested to determine the number of phages required to effectively lyse a single *V. parahaemolyticus* cell. Both VpP1 and VpP2 demonstrated a need for high MOIs (up to 1000,000) to achieve continuous suppression of bacterial growth, suggesting that elevated phage-to-host ratios may be necessary for effective biocontrol in post-harvest treatments. While both vibriophages showed similar trends, VpP2 demonstrated slightly better performance at lower MOIs compared to VpP1, suggesting it may be a more effective candidate for biocontrol. However, the requirement for such high MOIs is a significant limitation. Most studies have reported effective bacterial inhibition at much lower MOIs. For example, Cao et al. (2021) observed strong inhibition of *Vibrio* spp. at an MOI of 100, and other phages have shown efficacy at even lower ratios (Cao et al., 2021). The need for an MOI of 1000,000 in this study suggests that VpP1 and VpP2 may have relatively low infectivity or replication efficiency under the tested conditions. This could be due to factors such as low adsorption rates, small burst sizes, or host resistance mechanisms (Yan et al., 2024). Although it might seem appropriate to apply the highest possible phage dose for maximum bacterial removal, doing so may unintentionally increase selective pressure on the bacterial population, encouraging the emergence of resistant mutants (Nang et al., 2023). Understanding how different MOIs influence both killing efficiency and resistance development is essential for designing effective phage therapies (Torres-Barceló, 2018). Further studies should measure how effectively vibriophages VpP1 and VpP2 kill their host bacteria and whether resistance develops, both *in vitro* and *in vivo,* so they can be better applied to real-world applications. In addition, using the phages in combination or as part of a wider ‘cocktail’ may improve their efficiency.

The one-step growth curve experiment was used to examine the key phases of the vibriophage replication cycle, including the eclipse and latent periods, as well as the overall burst size (Louten, 2016). Both VpP1 and VpP2 exhibited first latent periods of approximately 22 min, followed by relatively small burst sizes of 11.8 and 8.6 PFU/cell, respectively. For comparison, typical phage burst sizes range from 50 to 200 PFU/cell (Louten, 2016). Smaller burst sizes often correlate with smaller plaque diameters (Fig. 1a and 1b), as fewer progeny phages are released per infected cell (Gallet et al., 2011). Although less common, low burst sizes have been reported in other vibriophages. For instance, Tan *et al*. (2021) observed burst sizes as low as 17 PFU/cell (Tan et al., 2021), while Chen *et al*. (2022) reported a burst size of 13 PFU/cell (Chen et al., 2022). From a therapeutic perspective, small burst sizes may limit the phage’s ability to rapidly reduce bacterial populations and reduce their ability to multiply and spread effectively at the site of infection. This could result in less efficient bacterial clearance and may require higher initial phage doses for effective treatment. While this may constrain biocontrol efficacy, such phages could still be valuable in combination therapies or in environments where slower propagation is advantageous.

Phage survivability is strongly influenced by external conditions such as pH, temperature, and salinity, which can affect their viability and effectiveness as biocontrol agents (Silva et al., 2014). Both vibriophages remained infective in salinity as low as 1 % and as high as 20 %, indicating their ability to potentially survive in brackish or marine waters. Li et al. (2022) demonstrated that phage vB_Vna-SL3 had a survival rate of approximately 88 % at ≤20 % salinity (Li et al., 2022). As brackish waters across Australia have an average salinity of 3.5 %, both phages have demonstrated their capability of surviving these environments (Dove and O'Connor, 2007).

VpP1 exhibited greater stability under basic conditions, while VpP2 tolerated more acidic environments. Both phages remained infective between pH 4–10, with VpP1 demonstrating limited survival even at pH 3. These findings align with previous studies, such as Cao et al. (2021), which reported optimal infectivity of *Vibrio*-specific phages between pH 3–9 (Cao et al., 2021). When phages are exposed to environmental pH levels that are lower than or equal to their isoelectric point (pI), they begin to aggregate, which can reduce their infectivity. This phenomenon explains the reduced activity of VpP1 and VpP2 observed at extreme pH values in this study. Aggregation limits phage mobility and impairs their ability to attach to and lyse bacterial cells, thereby diminishing their antibacterial effectiveness (Langlet et al., 2007). This characteristic also suggests that phages are less likely to disrupt the natural gut microbiota when consumed via phage-treated food, as the acidic conditions of the gastrointestinal tract may reduce their activity before they reach the intestines (Ly-Chatain, 2014). This property enhances their safety profile for potential use in food applications.

The temperature stability of the phages was tested, with both phages maintaining infectivity from −20 to 40 °C and becoming inactive at 80 °C. These results align with previous studies that tested *Vibrio*-specific phage stability at −20 to 70 °C (Cao et al., 2021; Tan et al., 2021).

To evaluate the stability of VpP1 and VpP2 under UV exposure, they were subjected to ultraviolet light for durations ranging from 30 s to 5 min. As UV radiation is increasingly adopted for water disinfection, its integration with phage-based treatments during post-harvest processes such as depuration has been proposed. However, both vibriophages showed a progressive decline in titre with each minute of exposure, and VpP1 was completely inactivated after 5 min. These findings align with those of Lomelí-Ortega et al. (2021), who reported a 50 % reduction in infectivity of their *Vibrio*-specific phage after just 60 s of UV exposure (Lomelí-Ortega et al., 2021). The results highlight the challenge of phage stability under UV exposure, particularly in the aquaculture system where UV treatment is routine. However, recent research by Wdowiak et al. (2025) demonstrated that the use of food dye, brilliant blue FCF, can enhance the UV resistance of non-enveloped phages, offering a potential strategy mitigate this limitation and improve phage stability during UV-based treatments (Wdowiak et al., 2025).

Free chlorine residue is often found in Australian waters. Additionally, oyster depuration is coupled with chlorine for increased bacterial elimination (Ramos et al., 2012). Since chlorine is a key component of sodium hypochlorite, this motivated testing VpP1 and VpP2 against varying concentrations with the results showing minimal variation in stability between 3 and 10 mg/L (Fig. 4e).

Bacteriophage-insensitive mutants (BIMs) are bacteria that naturally resist phage infection (Campbell, 1961). The observed cross-infectivity from Fig. 5 suggests that VpP1 and VpP2 likely target different bacterial receptors, allowing them to infect BIMs derived from alternate exposures. This indicates that resistance may be phage-specific rather than broad-spectrum. Although rare, reversion to sensitivity could occur under certain conditions. These findings highlight the importance of evaluating resistance dynamics when considering phage efficacy in therapeutic or biocontrol settings. Further studies are needed to assess the stability of BIM resistance and its implications for long-term phage efficacy (Wright et al., 2019).

To better understand the characteristics of both phages, whole-genome sequencing was performed. Genome analysis of VpP1 and VpP2 indicated linear, dsDNA genomes of 63,510 bp and 64,298 bp, respectively. The *G* + *C* content of 49 % for both phages corresponds to previously published reports on vibriophages genomes (Kim et al., 2021; Villa et al., 2012; Yonas et al., 2020). Many of the CDS annotated in both phage genomes have known, homologous functions based on previously reported studies. Notably, the RuvC-like Holliday junction resolvase aids in the in the creation of Holliday junctions which are derived from the exchange of single strands between two homologous DNA duplexes. Host bacteria carry this gene; however, phages use their own Holliday junction resolvase to ensure autonomy from the host cell’s machinery (Sharples, 2001). The enzyme, MazG-like pyrophosphatase, aids in phage survival by breaking down alarmones (bacterial stress signalling molecules). This signal normally triggers a self-destruct response in bacteria to stop phage infection. By removing it, the MazG enzyme prevents the bacteria from killing itself, allowing the phage to continue replicating (Ho et al., 2023). Historically, lysis of Gram-negative bacterial hosts required phages to carry the proteins holin, for the breakdown of the cytoplasmic membrane, and endolysins to degrade the host bacterium’s peptidoglycan layer. Recently, a third lysis protein was found to aid in the degradation of host bacteria, the spanins. Following the actions of holin and endolysins, the spanin protein is activated post-peptidoglycan degradation, where it disrupts the host outer membrane, facilitating phage exit from the cell (Cahill and Young, 2019). No tRNA encoding genes were found in either phage, indicating that both phages require the translation machinery of the host bacteria. No antibiotic-resistant or toxin genes were found upon genomic analysis. Additionally, the absence of the lysogenic gene supports regulatory approval for food use (Cristobal-Cueto et al., 2021).

In summary, this study presents the first detailed characterisation of the *Vibrio*-specific phages VpP1 and VpP2. Both vibriophages demonstrated the ability to reduce *V. parahaemolyticus* strains Vp60446 and Vp66674 *in vitro*, supporting their potential as biocontrol agents in aquaculture. However, their performance was not optimal, requiring unusually high MOIs and exhibiting relatively small burst sizes. These limitations may be partly attributed to the fact that the phages were originally isolated using different *Vibrio* strains that those used in this study, potentially affecting host-phage compatibility and lytic efficiency. Despite these challenges, the absence of toxin, lysogenic, or antibiotic resistance genes in their genomes supports their safety profile. While this study focuses on the characterisation of vibriophages, the importance of assessing their efficacy in live aquaculture environments is acknowledged. Ongoing trials of phage efficacy in real world applications, specifically oyster processing, are currently underway and will be reported separately. The results from the present study lay the groundwork for future *in vivo* testing for both aquaculture and food safety applications.

## Funding

This research was partially funded by the Fisheries Research and Development Corporation through the SafeFish program [2021–018].

## CRediT authorship contribution statement

**Madeline I. Petrusic:** Writing – original draft. **Sarah K. McLean:** Writing – review & editing. **Enzo A. Palombo:** Writing – review & editing.

## Declaration of competing interest

The authors declare that they have no known competing financial interests or personal relationships that could have appeared to influence the work reported in this paper.

## Data Availability

Data will be made available on request.
